# Mediterranean dietary pattern and risk of neurodegenerative diseases in a cohort of Swedish women

**DOI:** 10.1038/s41531-025-00932-1

**Published:** 2025-04-11

**Authors:** Emily E. Joyce, Weiyao Yin, Marie Löf, Karin Wirdefeldt, Sven Sandin, Fang Fang

**Affiliations:** 1 Institute of Environmental Medicine, Karolinska Institutet, Stockholm, Sweden; 2 Department of Medical Epidemiology and Biostatistics, Karolinska Institutet, Stockholm, Sweden; 3 Department of Biosciences and Nutrition, Karolinska Institutet, Stockholm, Sweden; 4 Department of Clinical Neuroscience, Karolinska Institutet, Stockholm, Sweden; 5 Department of Psychiatry, Icahn School of Medicine at Mount Sinai, New York, NY USA; 6 Seaver Autism Center for Research and Treatment at Mount Sinai, New York, NY USA

**Keywords:** Risk factors, Parkinson's disease

## Abstract

Mediterranean dietary patterns (MDP) may be neuroprotective. Using a large population-based cohort of 42,582 Swedish women, this study examined the association between MDP adherence and the risk of Parkinson’s disease (PD), Alzheimer’s disease (AD), and amyotrophic lateral sclerosis (ALS). During 1991–1992, women in the Women’s Lifestyle and Health Study reported dietary intake, and MDP adherence was calculated. Incident neurodegenerative diseases were identified using the Swedish National Patient Register through 2022. Women who reported high MDP adherence had a lower risk of PD (HR: 0.69, 95% CI: 0.49–0.95), primarily over age 60 (HR: 0.68, 95% CI: 0.47–0.97). A moderate-high MDP adherence was associated with a lower risk of ALS before age 60 (HR: 0.44, 95% CI: 0.19–0.99), but not overall. We observed no association between MDP adherence and AD. Our findings suggest higher adherence to a MDP may be protective against PD above age 60, and ALS before age 60.

## Introduction

The increasing prevalence of age-related neurodegenerative diseases, including Parkinson’s disease (PD), Alzheimer’s disease (AD), and amyotrophic lateral sclerosis (ALS)^1–3^, is a growing public health concern in an aging population^4^. This concern is further exacerbated by a lack of effective treatment options. The genetic predisposition to neurodegenerative diseases varies; although AD is more genetic^2,5^, only a small proportion of PD and ALS cases have a known genetic cause^6,7^. Nevertheless, there are potentially modifiable factors, including environmental and lifestyle factors, involved in the underlying mechanisms of all these diseases^5–7^. Understanding the role of modifiable risk factors is necessary for identifying potential preventive strategies to combat these diseases.

A Mediterranean dietary pattern (MDP) is suggested as a healthy diet pattern, particularly due to its anti-inflammatory and antioxidant properties^8,9^. This dietary pattern emphasizes a high consumption of fruits, vegetables, legumes, nuts, and olive oil, moderate-high intake of fish, moderate consumption of alcohol (traditionally red wine), and a low intake of meat and dairy^10^. A neuroprotective role has been proposed in relation to adherence to an MDP^11,12^. For instance, the intake of beneficial nutrients such as polyphenols and carotenes found in fruits and vegetables has been associated with reduced inflammatory biomarkers^13,14^. Polyphenols derived from olive oil have antioxidant properties that can mitigate oxidative stress^15,16^ and may have neuroprotective benefits such as enhanced cognition^17^. High dietary fiber that is emphasized in an MDP also has a favorable impact on the gut microbiota, where gut dysbiosis has been associated with several neurodegenerative diseases^18,19^. In previous prospective cohort studies, adherence to an MDP has been shown to be associated with a lower risk of PD^20–22^ and AD^23–26^, though some of these studies suffer from small sample sizes^22–26^ and short follow-up times^23–26^. The association between MDP and ALS has not yet been established.

This study used dietary data reported during the six months before cohort entry in the Women’s Lifestyle and Health (WLH) Study in the early 1990s, consisting of almost 50,000 women aged 29 to 49 living in Uppsala, Sweden^27^. In a previous study using follow-up data until December 31, 2012, we found that a higher adherence to an MDP was associated with a reduced risk of PD^20^. With 10 additional years of follow-up, this study aimed to reproduce the finding in PD and compare whether the association would differ between PD and other neurodegenerative diseases, namely AD and ALS, in the same population.

## Results

### Baseline characteristics

Of the 49,260 women aged 29–49 enrolled in the WLH study, we excluded those who had emigrated prior to the cohort entry (n = 1,061), had no response in the FFQ (n = 567), reported extreme energy intake ( < 1^st^ or >99^th^ percentile in the cohort) (n = 604), or had prevalent disease (PD, AD, or ALS) (n = 6) at cohort entry (Supplemental Fig. 1). Missingness in the covariates was low ( <10%), and study participants with missing information on any of the covariates (n = 4440) were excluded for a complete case analysis, which resulted in 42,582 women eligible to be included in the analysis. Characteristics of the women excluded due to missing information on the covariates are described in Supplemental Table 1. Median consumption of the nine food groups by MDP adherence is shown in Supplemental Table 2.

**Fig. 1:**
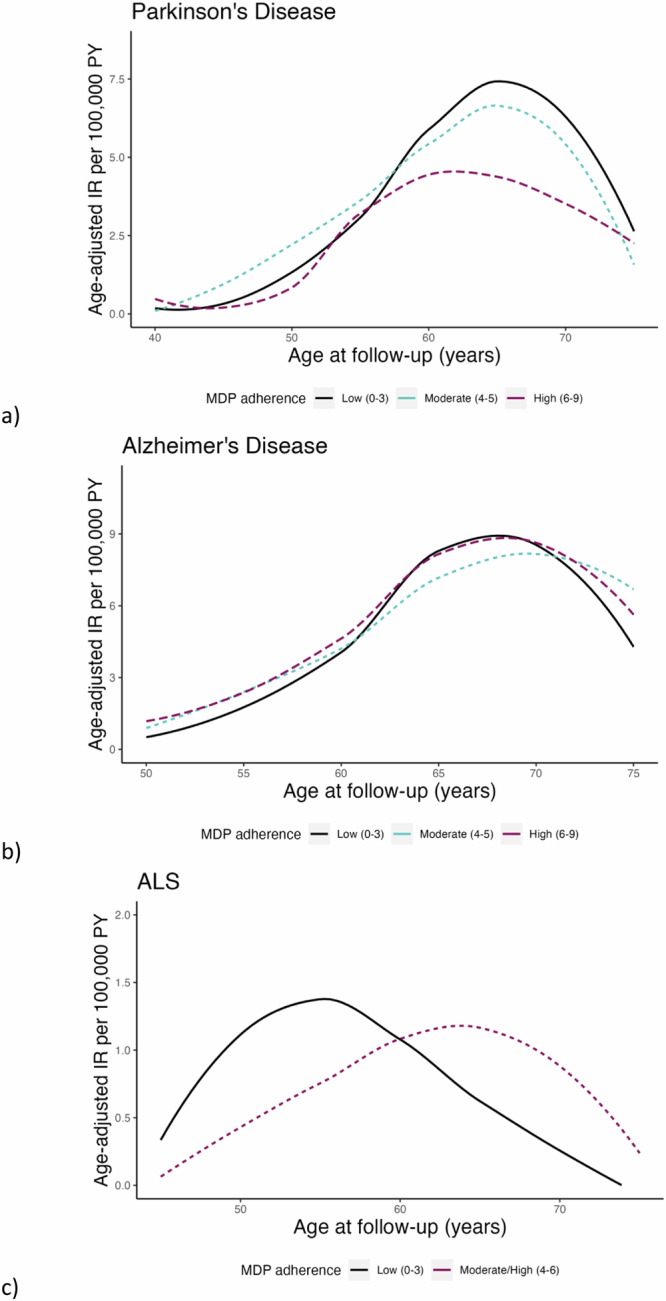
Age-adjusted incidence rates by Mediterranean dietary pattern (MDP) adherence over age at follow-up. Age-adjusted incidence rates of **a** Parkinson’s disease (PD), **b** Alzheimer’s disease (AD), and **c** amyotrophic lateral sclerosis (ALS) by different levels of Mediterranean dietary pattern (MDP) adherence over age at follow-up. Age-standardized IR were calculated in 5-year age intervals by MDP adherence, and a loess smoothing curve was used to model the shape of the IR over age at follow-up. IR incidence rate, PY person-years.

Summary statistics indicate that women who had a high adherence to an MDP at baseline tended to be older, more educated, had a higher level of physical activity, and more likely not to smoke, compared with women who had a low adherence (Table 1). In the average 29 years of follow-up, 305 women were diagnosed with PD (age-adjusted IR: 24.7 per 100,000 person-years), 368 women were diagnosed with AD (age-adjusted IR: 29.8 per 100,000 person-years), and 59 women were diagnosed with ALS (age-adjusted IR: 4.8 per 100,000 person-years). The age-adjusted IR by MDP adherence visualized over age at follow-up suggested a lower incidence of PD among women with a high adherence to MDP after age 60, and a lower incidence of ALS among women with moderate-high adherence before age 60 (Fig. 1).

**Table 1:** Baseline characteristics of study cohort by Mediterranean dietary pattern adherence

	Adherence to Mediterranean dietary pattern^a^, *N* (%)		
Characteristics	Low (0–3) *N* = 14,473	Moderate (4–5) *N* = 18,571	High (6–9) *N* = 9538
Age at enrollment (years)			
29–34	4033 (27.9)	4338 (23.4)	1826 (19.1)
35–39	3832 (26.5)	4829 (26.0)	2295 (24.1)
40–44	3537 (24.4)	4976 (26.8)	2717 (28.5)
45–49	3071 (21.2)	4428 (23.8)	2700 (28.3)
Birth year			
1942–1946	3196 (22.1)	4590 (24.7)	2799 (29.3)
1947–1951	3521 (24.3)	4927 (26.5)	2674 (28.0)
1952–1956	3851 (26.6)	4862 (26.2)	2323 (24.4)
1957–1962	3905 (27.0)	4192 (22.6)	1742 (18.3)
Body mass index (kg/m^2^)			
<25	10,469 (72.3)	13,278 (71.5)	7005 (73.4)
25–30	3106 (21.5)	4191 (22.6)	2084 (21.8)
≥30	898 (6.2)	1102 (5.9)	449 (4.7)
Education, years			
0–10	4954 (34.2)	5356 (28.8)	2439 (25.6)
11–13	5863 (40.5)	7212 (38.8)	3494 (36.6)
>13	3656 (25.3)	6003 (32.3)	3605 (37.8)
Physical activity			
Very low	813 (5.6)	695 (3.7)	257 (2.7)
Low	1734 (12.0)	1967 (10.6)	856 (9.0)
Moderate	8870 (61.3)	11,096 (59.7)	5434 (57.0)
High	2077 (14.4)	3225 (17.4)	1965 (20.6)
Very high	979 (6.8)	1588 (8.6)	1026 (10.8)
Diabetes			
No	14,282 (98.7)	18,320 (98.6)	9405 (98.6)
Yes	191 (1.3)	251 (1.4)	133 (1.4)
Hypertension			
No	13,147 (90.8)	16,808 (90.5)	8644 (90.6)
Yes	1326 (9.2)	1763 (9.5)	894 (9.4)
Smoking			
Never	5505 (38.0)	7844 (42.2)	4082 (42.8)
Former	5293 (36.6)	6876 (37.0)	3789 (39.7)
Current	3675 (25.4)	3851 (20.7)	1667 (17.5)
Total energy intake (kJ/day)^b^	6160 (1850)	6610 (1880)	6940 (1770)

^a^MDP score, calculated through self-reported food frequency questionnaires, was categorized into low (score 0–3), moderate (4–5), and high (6–9) adherence.

^b^Mean (standard deviation).

### Adherence to MDP and risk of neurodegenerative diseases

Compared with a low adherence, women with a high adherence to an MDP had a 31% reduced risk of PD in the fully adjusted model over the entire follow-up (HR = 0.69, 95% CI = 0.49–0.95) (Supplemental Table 3). The spline regression showed a linear fit to the association (Supplemental Fig. 2). There was no overall association observed between MDP adherence and risk of AD or ALS, when studying adherence as either a categorical or a continuous variable.

After visual inspection of the Schoenfeld residuals, which suggested non-proportionality of the hazards over time (Supplemental Fig. 3), we stratified the analysis by attained age at 60 years. The association of a high adherence to an MDP with a lower risk of PD was only noted after 60 years of age (HR = 0.68, 95% CI = 0.47–0.97), but not before (HR = 0.73, 95% CI = 0.22–1.50) (Table 2). There was no association observed between MDP adherence and AD when stratified by age (below or above 60). However, a moderate-high adherence to MDP (HR = 0.44, 95% CI = 0.19–0.98) and a one-unit increase in MDP adherence (HR = 0.77, 95% CI = 0.59–0.99) was associated with a lower risk of ALS at age 60 or below, but not above (Table 2).

**Table 2:** Association between Mediterranean dietary pattern (MDP) adherence and risk of Parkinson’s disease (PD) and Alzheimer’s disease (AD) and amyotrophic lateral sclerosis (ALS), stratified analysis by age at follow-up <60 and ≥60 years

Adherence to MDP	Cases/100,000 PY (IR)	Age-adjusted IR	Minimally adjusted HR (95% CI)^a^	Fully adjusted HR (95% CI)^b^
PD				
Age at follow-up < 60 years				
Low (0–3)	21/2.92 (7.2)	7.4	*Reference*	*Reference*
Moderate (4–5)	38/3.62 (10.5)	10.5	1.43 (0.85–2.48)	1.32 (0.78–2.30)
High (6–9)	11/1.79 (6.1)	5.9	0.82 (0.38–1.67)	0.73 (0.33–1.50)
Per unit increase	70/8.33 (8.4)		1.00 (0.87–1.16)	0.98 (0.84–1.13)
Age at follow-up ≥60 years				
Low (0–3)	85/1.28 (66.4)	67.8	*Reference*	*Reference*
Moderate (4–5)	103/1.77 (58.2)	58.2	0.86 (0.64–1.14)	0.85 (0.63–1.13)
High (6–9)	47/0.98 (47.9)	47.0	**0.68 (0.47–0.97)**	**0.68 (0.47–0.97)**
Per unit increase	235/4.03 (58.3)		**0.90 (0.83–0.97)**	**0.90 (0.83–0.97)**
AD				
Age at follow-up < 60 years				
Low (0–3)	10/2.92 (3.4)	3.5	*Reference*	*Reference*
Moderate (4–5)	19/3.62 (5.2)	5.2	1.49 (0.71–3.34)	1.43 (0.67–3.21)
High (6–9)	11/1.79 (6.1)	5.9	1.72 (0.72–4.15)	1.61 (0.66–3.94)
Per unit increase	40/8.33 (4.8)		1.13 (0.93–1.37)	1.11 (0.91–1.35)
Age at follow-up ≥60 years				
Low (0–3)	98/1.28 (76.5)	79.1	*Reference*	*Reference*
Moderate (4–5)	145/1.77 (81.9)	81.9	1.03 (0.80–1.33)	1.03 (0.79–1.33)
High (6–9)	85/0.98 (86.6)	82.7	1.04 (0.77–1.38)	1.02 (0.76–1.38)
Per unit increase	328/4.04 (81.3)		1.01 (0.95–1.08)	1.01 (0.94–1.08)
ALS				
Age at follow-up < 60 years				
Low (0–3)	14/2.92 (4.8)	4.9	*Reference*	*Reference*
Moderate-High (4–9)	11/5.41 (2.0)	2.0	**0.40 (0.18–0.89)**	**0.44 (0.19–0.98)**
Per unit increase	25/8.33 (3.0)		**0.75 (0.59–0.96)**	**0.77 (0.59–0.99)**
Age at follow-up ≥60 years				
Low (0–3)	6/1.29 (4.7)	4.6	*Reference*	*Reference*
Moderate-High (4–9)	28/2.76 (10.1)	10.1	2.14 (0.95–5.73)	1.81 (0.79–4.89)
Per unit increase	34/4.05 (8.4)		**1.29 (1.04–1.59)**	1.22 (0.98–1.52)

*PY* person-years, *IR* incidence rate per 100,000 person-years, *HR* hazard ratio, *CI* confidence interval, *BMI* body mass index.

^a^HR and 95% CI were estimated using Cox model with attained age as the underlying time scale and adjustment for year of birth (1942–1946, 1947–1951, 1952–1956 and 1957–1962).

^b^HR and 95% CI were estimated using Cox model with attained age as the underlying time scale and adjustment for year of birth, BMI, education, physical activity, smoking status, medical history of diabetes and hypertension, and total energy intake.

Bold values are statistically significant.

### Sensitivity analysis

Removing the first two or five years of follow-up showed similar results (Supplemental Table 4).

## Discussion

Leveraging nearly 30 years of follow-up in a large cohort of Swedish women, we found that a higher adherence to an MDP at age 29–49 was associated with a lower risk of PD above age 60. The finding on PD is consistent with results from our previous study with a shorter follow-up time^20^. Overall, no association was observed between MDP adherence and AD. However, a higher adherence to this MDP was found to be associated with a lower risk of ALS up until approximately age 60 or below.

The impact of MDP adherence on PD risk has been described in other observational studies, some of which have suggested a protective effect of an MDP^20–22,28,29^, while others reported null associations^30,31^. Several of these studies were cross-sectional and compared the dietary intake of prevalent PD cases with controls^28–30^. Since diet was assessed after PD diagnosis, the conclusions drawn in these cross-sectional studies cannot be extended to PD risk. Beyond the MDP, high consumption of dairy products has been suggested as a risk factor for PD^32^, which is considered a non-beneficial food group in an MDP. Other studies have investigated the role of individual nutrients, such as antioxidants and dietary fat, with PD risk, most of which have shown inconsistent associations^33,34^. In the WLH Study, we observed a protective effect of this MDP with PD risk above age 60. The diminished protective association for PD before age 60, compared to after age 60, was perhaps driven by a stronger genetic involvement in early-onset PD^35^, or from a lack of precision due to a smaller number of diagnoses before 60 years.

On the other hand, there was no association observed between MDP and AD in the present study. Similar to our findings, a prospective cohort study in Swedish men showed no association between MDP and AD risk with 12 years of follow-up^36^. However, there is a lack of consistent findings reported in other prospective cohort studies^23–26,36,37^. Reverse causality may be a main limitation in these studies, due to the presumably long preclinical stage of AD. Many previous studies had a short follow-up time between dietary exposure and disease onset ( <6 years)^23–26^, and dietary intake was assessed in a population older than 70 years^23–26,36^. Comparison between these studies may be further complicated due to the heterogeneity of the diagnostic criteria of AD used in different studies.

Thus far, associations between an MDP and ALS have not been studied. In this study, we observed a higher adherence to an MDP was associated with a reduced risk of ALS at 60 or younger, but not above age 60. However, the small number of cases identified contributed to low power in this analysis, leaving random error a potential concern. Although body composition and energy homeostasis have been associated with ALS risk^7,38^, the association between individual food and nutrient intake and ALS is largely unknown^39–42^. Larger prospective studies are needed to validate our findings on both ALS and PD, to understand whether the noted age-and disease-specific associations are true.

An advantage to studying MDP adherence as a dietary pattern, rather than individual foods or nutrients, is the ability to account for the interactions between consumed foods and nutrients, and the cumulative effects of a diet over a long period of time^43^. However, it is important to consider that a healthy diet is often associated with other healthy lifestyle behaviors, such as increased physical activity and reduced smoking^44^, and these health-promoting factors have been shown to reduce all-cause mortality in an additive manner^45^. In order to account for the complicated relationship between diet and other lifestyle factors, we adjusted for a wide range of variables that could be confounders in the association between diet and the three neurodegenerative diseases that we studied, although residual confounding may remain.

This study has many strengths, notably the inclusion of a large cohort of Swedish women who had complete dietary data and reported demographic information, and the ability to follow these women for almost 30 years, with virtually no loss to follow-up due to the completeness of the Swedish National Patient Register. However, there are several limitations of this study. We only had one dietary measurement using the FFQ at baseline, which evaluated dietary intake in the six months before cohort entry, so we could not evaluate changes in MDP adherence during follow-up. Since the MDP used in this study was based on the median intake of this cohort of Swedish women, the conclusions made using this MDP may not be generalizable to other populations that have a different dietary intake. However, it is relevant to note that the median intakes of the food groups used to calculate the MDP in the WLH cohort are comparable to the corresponding ones from the most recent national survey in Sweden^46^. Additionally, since neurodegenerative diseases were defined through the Swedish National Patient Register based on inpatient or outpatient hospital visits, we had no additional information about disease characteristics, including genetic causes. Therefore, no conclusion could be made about the possible influence of genetic factors in the observed associations. Further, in our study most women had follow-up data into their 70 s, and incidence for neurodegenerative diseases steadily increases with age. Since more women in the WLH Study may be diagnosed with PD, AD, or ALS in the coming years, a follow-up study is necessary. Finally, since the WLH study only recruited women, our findings might not directly extend to men.

It has been observed that the consumption of certain foods and food components may pose a risk to one’s health^47,48^. Considering the steady increase in the prevalence of neurodegenerative diseases, it is important to understand whether a healthy diet can reduce the risk of these diseases to inform public health interventions and prevention for at-risk groups. To fully understand how an MDP is associated with neurodegeneration, future studies should evaluate dietary changes that occur over the lifetime, investigate the interactions between dietary intake and other lifestyle factors, and measure underlying biological processes.

Higher adherence to a Mediterranean dietary pattern may be protective against Parkinson’s disease above age 60. The observed association between a moderate-high adherence to a Mediterranean dietary pattern and a reduced risk of ALS before age 60 is however novel and needs to be validated in future studies.

## Methods

Between August 1991 and March 1992, 96,000 women aged 29 to 49 years residing in the Uppsala region in Sweden were randomly selected from the Swedish Total Population Register and invited to participate in the WLH Study^27^. Among those invited, 49,260 (51%) women consented to be a part of this study, and returned the baseline questionnaire, which included a food frequency questionnaire (FFQ). The median age at enrollment was 40 years. Follow-up began at the date of returning the baseline questionnaire. We followed women until the date of PD, AD, or ALS diagnosis, emigration from Sweden, death, or March 31^st^, 2022, whichever occurred first. We used the Swedish National Patient Register to identify incident cases of PD, AD, and ALS, and the Total Population Register to identify emigration and death, through cross-linking the WLH Study and these registers using unique Swedish personal identity numbers. The study was approved by the Swedish Ethical Review Authority (DNR: 2020-02976). Informed consent to participate in the study was collected from all study participants.

### Exposure assessment

Participants were asked to recall the frequency and quantity of 80 foods and beverages consumed over the previous six months before cohort entry. The total consumption (g/day) of foods and drinks were summarized, and intakes of energy (kJ/day), and nutrients were calculated by linkage to the Swedish National Food Administration database^49^. Further, we calculated adherence to an MDP using the scale developed by Trichopoulou et al.^10^ as in other studies using the WLH Study^20,50^.

An MDP was calculated based on the following nine food groups: vegetables, fruits and nuts, cereals, legumes, dairy products, fish and seafood, meat, alcohol, and monounsaturated-to-saturated (M/S) fat ratio. All participants were scored on each food group based on the median consumption of the entire cohort (Supplemental Table 5). Consumption greater than or equal to the cohort median for beneficial food groups, including vegetables, fruits and nuts, cereals, legumes, fish and seafood, and high M/S fat ratio, was scored as 1, where consumption less than the cohort median was scored 0. Conversely, non-beneficial food groups, dairy products and meat, were scored in the reverse direction. Alcohol was scored separately, where moderate level of consumption (5–25 g/day) was scored as 1, and greater than or less than moderate consumption was scored 0. Scores among all nine food groups were summed, yielding a score of MDP adherence ranging from 0 (least adherent) to 9 (most adherent). This score was also categorized into low (score 0–3), moderate (4–5), and high (6–9) adherence^10^.

### Outcome assessment

The Swedish National Patient Register was used to identify the first clinical diagnosis of the studied neurodegenerative diseases, where nationwide data on inpatient care was available from 1987 and outpatient care was available from 2001^51^. The following codes of the Swedish revisions of the International Classification of Disease (ICD) were used to define PD (ICD-9: 332 A, 333 A, ICD-10: G20, F023, G214, G218, G219, G231, G232, G239, G259, G318A), AD (ICD-9: 290 A, 290B, 331 A, ICD-10: F00, G30), and ALS (ICD-9: 335 C, ICD-10: G12.2). Using ICD codes to identify neurodegenerative diseases based on the National Patient Register has been validated against clinical diagnosis, demonstrating high specificity for PD ( > 98%)^52^ and AD (99.7%)^53^, and a high positive predictive value for ALS (91%)^54^.

### Covariates

Questionnaires administered at baseline also collected information on demographic characteristics, lifestyle factors, and medical history. We included several variables as potential confounders in the association between MDP and PD, AD, and ALS, including year of birth (1942–1946, 1947–1951, 1952–1956, 1957–1962), body mass index (BMI in kg/m^2^, <25, ≥25 and <30, ≥30), years of education (0–10, 11–13, >13), level of physical activity (self-reported levels: very low, low, moderate, high, very high), smoking status (never, former, current), and total energy intake (kJ/day) calculated through the Swedish National Food Administration database^49^. Since the presence of a morbidity may influence dietary intake and the future risk for neurodegenerative diseases, we additionally included self-reported medical history of diabetes or hypertension (yes, no) at baseline as covariates.

### Statistical analysis

Age-adjusted incidence rates (IR) were calculated for PD, AD, and ALS separately, standardized using the distribution of attained age during the follow-up of the entire cohort. Additionally, age-adjusted IR for PD, AD, and ALS were calculated in 5-year age intervals by MDP adherence and visualized using a loess smoothing curve over age at follow-up. The association between MDP and PD, AD, and ALS was assessed using Cox regression models to estimate the hazard ratios (HRs) and 95% confidence intervals (95% CIs), using attained age (age at follow-up) as the underlying time scale. This MDP was evaluated as both a categorical (0–3 low, 4–5 moderate, 6–9 high) and continuous (0–9, per unit increase) variable. In a minimally adjusted model, we adjusted for year of birth, and in the fully adjusted model we additionally adjusted for BMI, education, physical activity, smoking status, history of diabetes and hypertension, and total energy intake as defined above. Natural cubic splines were fitted to assess the risk of PD, AD, and ALS across MDP score as a continuous variable (0–9), using an MDP score 4 as the reference. The proportional hazards assumption was assessed through the standardized Schoenfeld residuals for each outcome.

Since older age is a common risk factor across these neurodegenerative diseases, we fitted the Cox regression models in sub-groups of attained age <60 and ≥60 years according to the findings of age-adjusted IR over age at follow-up and upon evaluation of the Schoenfeld residuals. In the age-stratified analysis of ALS, we collapsed the high and moderate adherence MDP levels to account for reduced numbers of ALS cases.

To alleviate the concern of potential reverse causation, we removed the first two or the first five years of follow-up from the analysis in a sensitivity analysis.

Statistical analyses were performed using SAS software version 9.4 (SAS Institute Inc., Cary, NC). All tests of statistical hypotheses were done on the two-sided 5% level of significance. This study followed the STROBE (Strengthening the reporting of observational studies in epidemiology) guidelines for cohort studies (Supplemental Table 6).

## Supplementary information

Supplementary information

## Data Availability

Data from the WLH Study are available to qualified investigators upon request. For more information on the WLH Study please visit: https://maelstrom-research.org/study/swlh. The SAS code for this study may be made available to qualified researchers on reasonable request from the corresponding author.
