# Supplementary material for: Mediterranean dietary pattern and risk of neurodegenerative diseases in a cohort of Swedish women
Source: NPJ Parkinsons Dis. 2025 Apr 11;11:71. doi: 10.1038/s41531-025-00932-1 (PMC11986014; doi:10.1038/s41531-025-00932-1)
Supplement: Supplementary file 1 — Supplementary information [file 41531_2025_932_MOESM1_ESM.docx]

**Mediterranean Dietary Pattern and Risk of Neurodegenerative Diseases in a Cohort of Swedish Women**

*npj Parkinson's Disease*

Emily E. Joyce^1+^, Weiyao Yin^2^, Marie Löf^3^, Karin Wirdefeldt^2,4^, Sven Sandin^2,5,6^, Fang Fang^1^

1. Institute of Environmental Medicine, Karolinska Institutet, Stockholm, Sweden

2. Department of Medical Epidemiology and Biostatistics, Karolinska Institutet, Stockholm, Sweden

3. Department of Biosciences and Nutrition, Karolinska Institutet, Stockholm, Sweden

4. Department of Clinical Neuroscience, Karolinska Institutet, Stockholm, Sweden

5. Department of Psychiatry, Icahn School of Medicine at Mount Sinai, New York, USA

6. Seaver Autism Center for Research and Treatment at Mount Sinai, New York, USA

+Corresponding Author:

Emily E. Joyce, MS

emily.joyce@ki.se

Supplemental Figure 1: Flow chart of exclusion criteria and analysis cohort

Women’s Lifestyle and Health Cohort

Recruitment: 1991-1992

Women aged 29-49

Uppsala Health Care Region

Consented to participate: 49,260

Followed until disease, emigration, death, or March 31^st^, 2022

Missing data on covariates: 4,440

Excluded from study: 2,238

- Emigrated prior: 1,061

- No response in FFQ: 567

- Extreme energy intake: 604

- Prevalent PD, AD, ALS: 6

Participants reported on dietary intake, lifestyle, and demographic characteristics through questionnaires

PD: n=305

AD: n=368

ALS: n=59

Analysis cohort: **42,582**

Supplemental Table 1: Baseline characteristics of women excluded due to missing value on the main covariates by adherence to the Mediterranean dietary pattern

|  | Adherence to Mediterranean Dietary Pattern, N (%) | | |
| --- | --- | --- | --- |
| Characteristics | **Low (0-3)**  N=1,663 | **Moderate (4-5)**  N=1,865 | **High (6-9)**  N=912 |
| Age at enrolment (years) |  |  |  |
| 29-34 | 833 (50.1) | 816 (43.8) | 377 (41.3) |
| 35-39 | 264 (15.9) | 329 (17.6) | 145 (15.9) |
| 40-44 | 247 (14.9) | 302 (16.2) | 163 (17.9) |
| 45-49 | 319 (19.2) | 418 (22.4) | 227 (24.9) |
| Body mass index (kg/m^2^) |  |  |  |
| <25 | 793 (47.7) | 826 (44.3) | 409 (44.8) |
| 25-30 | 184 (11.1) | 203 (10.9) | 112 (12.3) |
| ≥30 | 60 (3.6) | 63 (3.4) | 30 (3.3) |
| *Missing* | 626 (37.6) | 773 (41.4) | 361 (39.6) |
| Education, years |  |  |  |
| 0-10 | 449 (27.0) | 408 (21.9) | 170 (18.6) |
| 11-13 | 618 (37.2) | 629 (33.7) | 296 (32.5) |
| >13 | 292 (17.6) | 439 (23.5) | 217 (23.8) |
| *Missing* | 304 (18.3) | 389 (20.9) | 229 (25.1) |
| Physical activity |  |  |  |
| Very low | 73 (4.4) | 68 (3.6) | 30 (3.3) |
| Low | 96 (5.8) | 101 (5.4) | 43 (4.7) |
| Moderate | 517 (31.1) | 635 (34.0) | 316 (34.6) |
| High | 89 (5.4) | 163 (8.7) | 89 (9.8) |
| Very high | 42 (2.5) | 73 (3.9) | 55 (6.0) |
| *Missing* | 846 (50.9) | 825 (44.2) | 379 (41.6) |
| Diabetes |  |  |  |
| No | 1635 (98.3) | 1836 (98.4) | 903 (99.0) |
| Yes | 28 (1.7) | 29 (1.6) | 9 (1.0) |
| Hypertension |  |  |  |
| No | 1505 (90.5) | 1682 (90.2) | 825 (90.5) |
| Yes | 158 (9.5) | 183 (9.8) | 87 (9.5) |
| Smoking |  |  |  |
| Never | 652 (39.2) | 807 (43.3) | 415 (45.5) |
| Former | 648 (39.0) | 728 (39.0) | 366 (40.1) |
| Current | 363 (21.8) | 330 (17.7) | 131 (14.4) |

MDP score, calculated through self-reported food frequency questionnaires, was categorized into low (score 0-3), moderate (4-5), and high (6-9) adherence.

N=4,440 participants were excluded due to missing value in model covariates

Supplemental Table 2: Consumption of different components of Mediterranean dietary pattern in the Women’s Lifestyle and Health cohort by Mediterranean dietary pattern adherence

| Component | Consumption (Median, g/d) | | |
| --- | --- | --- | --- |
|  | **Low (0-3)**  N=14,473 | **Moderate (4-5)**  N=18,571 | **High (6-9)**  N=9,538 |
| Vegetables | 41.89 | 67.00 | 87.25 |
| Fruits and nuts | 94.84 | 147.50 | 195.68 |
| Legumes | 6.00 | 17.54 | 26.20 |
| Cereals | 158.90 | 187.67 | 215.19 |
| Fish and seafood | 16.39 | 24.46 | 31.11 |
| Meat | 85.69 | 85.21 | 81.64 |
| Dairy products | 429.30 | 310.54 | 232.77 |
| M/S ratio | 0.70 | 0.76 | 0.81 |
| Alcohol | 1.33 | 1.85 | 2.81 |

M/S: Monounsaturated fat/saturated fat

Supplemental Table 3: Association between Mediterranean dietary pattern (MDP) adherence and the risk of Parkinson’s disease (PD), Alzheimer’s disease (AD), and amyotrophic lateral sclerosis (ALS)

| Adherence to MDP | Cases/100,000 PY (IR) | Age-adjusted IR | Minimally adjusted HR (95% CI) ^a^ | Fully adjusted HR  (95% CI) ^b^ |
| --- | --- | --- | --- | --- |
| **PD** |  |  |  |  |
| Low (0-3) | 106/4.20 (25.2) | 27.1 | *Reference* | *Reference* |
| Moderate (4-5) | 141/5.39 (26.2) | 26.0 | 0.96 (0.75-1.24) | 0.94 (0.73-1.21) |
| High (6-9) | 58/2.77 (20.9) | 19.3 | **0.71 (0.51-0.97)** | **0.69 (0.49-0.95)** |
| Per unit increase | 305/12.36 (24.7) |  | **0.92 (0.86-0.99)** | **0.91 (0.85-0.98)** |
| **AD** |  |  |  |  |
| Low (0-3) | 108/4.20 (25.7) | 28.2 | *Reference* | *Reference* |
| Moderate (4-5) | 164/5.39 (30.4) | 30.2 | 1.07 (0.84-1.37) | 1.06 (0.83-1.36) |
| High (6-9) | 96/2.77 (34.6) | 30.9 | 1.09 (0.83-1.44) | 1.07 (0.81-1.42) |
| Per unit increase | 368/12.36 (29.8) |  | 1.02 (0.96-1.09) | 1.02 (0.96-1.09) |
| **ALS** |  |  |  |  |
| Low (0-3) | 20/4.20 (4.8) | 4.8 | *Reference* | *Reference* |
| Moderate (4-5) | 24/5.40 (4.4) | 4.4 | 0.89 (0.49-1.63) | 0.85 (0.47-1.57) |
| High (6-9) | 15/2.78 (5.4) | 5.0 | 1.03 (0.52-2.02) | 0.94 (0.46-1.87) |
| Per unit increase | 59/12.38 (4.8) |  | 1.03 (0.88-1.20) | 1.00 (0.85-1.18) |

Abbreviations: PY, person-years; IR, incidence rate per 100,000 person-years. HR, hazard ratio; CI, confidence interval; BMI, body mass index

^a^ HR and 95% CI were estimated using Cox model with attained age as the underlying time scale and adjustment for year of birth (1942-46, 1947-51, 1952-56 and 1957-62)

^b^ HR and 95% CI were estimated using Cox model with attained age as the underlying time scale and adjustment for year of birth, BMI, education, physical activity, smoking status, medical history of diabetes and hypertension, and total energy intake

Supplemental Figure 2: Adjusted hazard ratios and 95% confidence intervals of a) Parkinson’s disease (PD), b) Alzheimer’s disease (AD), and c) amyotrophic lateral sclerosis (ALS) by adherence to Mediterranean dietary pattern (MDP), referenced on an adherence score of 4, using natural cubic splines^a^

a
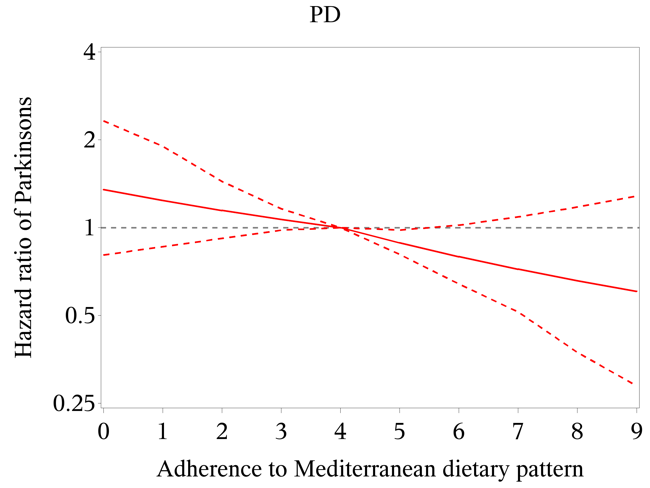
 b
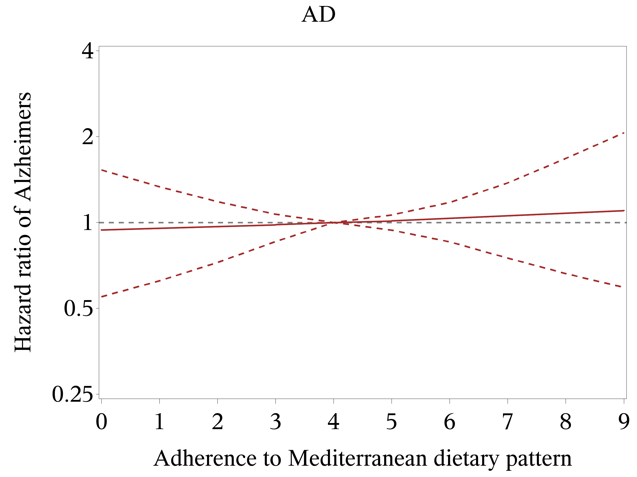


c
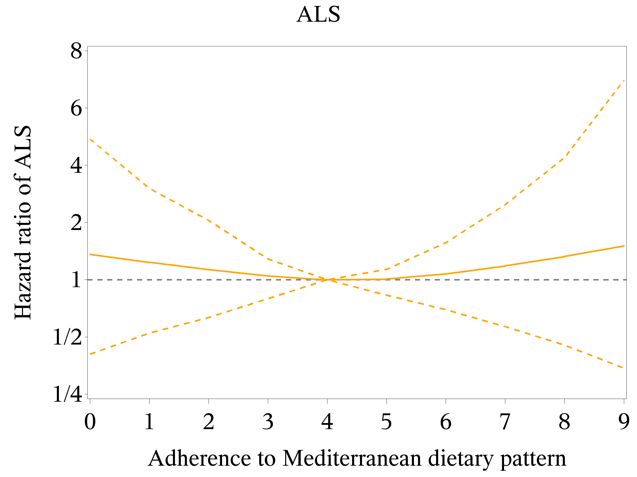


^a^ Adjusted for year of birth, body mass index, education, physical activity, smoking status, medical history of diabetes and hypertension, and total energy intake

Supplemental Figure 3: Test of the assumption of proportional hazards by scaled Schoenfield residuals, comparing high adherence (6-9) to low adherence (0-3) of the Mediterranean dietary pattern (MDP), in a) Parkinson’s disease (PD), b) Alzheimer’s disease (AD), and c) amyotrophic lateral sclerosis (ALS)

a
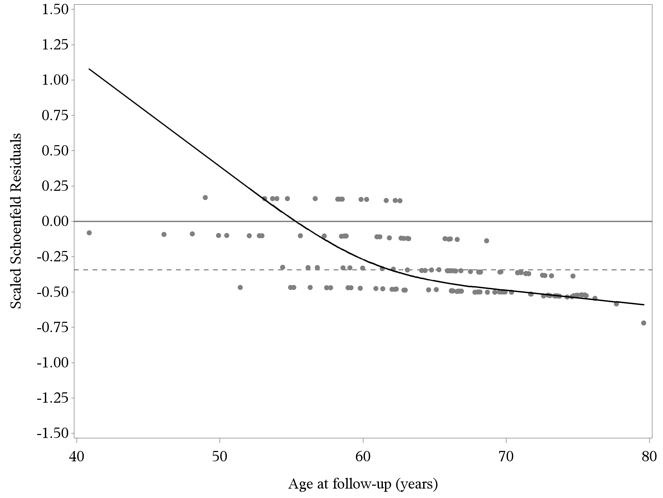
 b
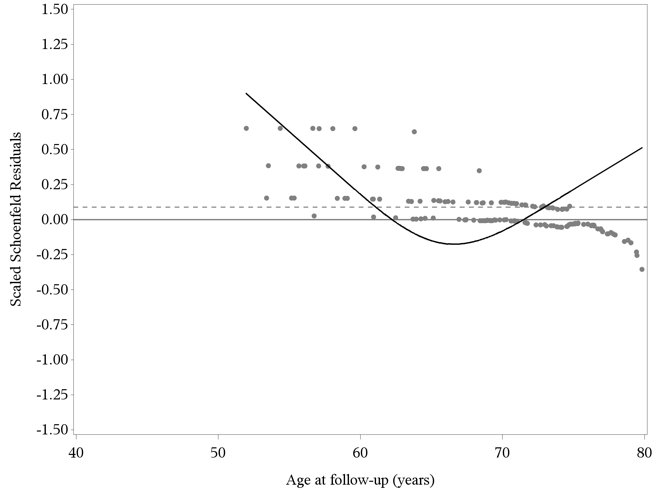


c
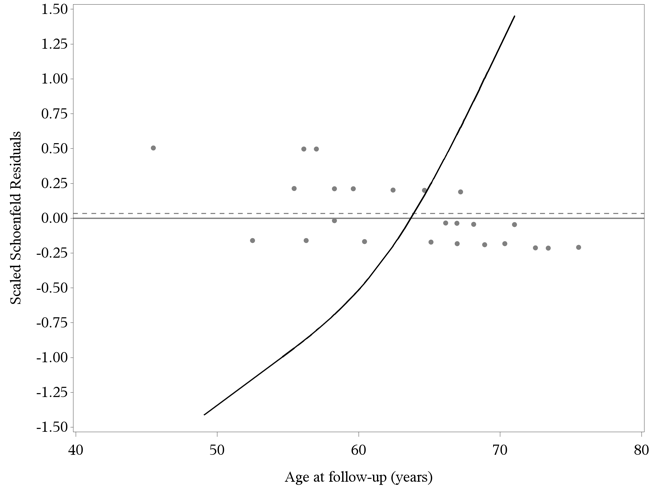


Supplemental Table 4: Association between adherence to Mediterranean dietary pattern (MPD) and risk of Parkinson’s disease (PD), Alzheimer’s disease (AD), and amyotrophic lateral sclerosis (ALS) after removing the first 2 years or the first 5 years of follow-up

| Adherence to MDP | Cases/100,000 PY (IR) | Excluding first 2 years of follow-up  HR (95% CI)^a^ | Cases/100,000 PY (IR) | Excluding first 5 years of follow-up  HR (95% CI)^a^ |
| --- | --- | --- | --- | --- |
| **PD** |  |  |  |  |
| Low (0-3) | 106/3.91 (27.1) | *Reference* | 105/3.47 (30.2) | *Reference* |
| Moderate (4-5) | 141/5.02 (28.1) | 0.94 (0.73-1.21) | 140/4.46 (31.4) | 0.94 (0.73-1.22) |
| High (6-9) | 58/2.58 (22.5) | **0.69 (0.49-0.95)** | 58/2.30 (25.2) | **0.69 (0.50-0.96)** |
| Per unit increase | 305/11.51 (26.5) | **0.91 (0.85-0.98)** | 303/10.24 (29.6) | **0.92 (0.85-0.98)** |
| **AD** |  |  |  |  |
| Low (0-3) | 108/3.91 (27.6) | *Reference* | 108/3.48 (31.0) | *Reference* |
| Moderate (4-5) | 164/5.02 (32.7) | 1.06 (0.83-1.36) | 164/4.47 (36.7) | 1.06 (0.83-1.36) |
| High (6-9) | 96/2.58 (37.2) | 1.07 (0.81-1.42) | 96/2.30 (41.7) | 1.07 (0.81-1.42) |
| Per unit increase | 368/11.51 (32.0) | 1.02 (0.96-1.09) | 368/10.25 (35.9) | 1.02 (0.96-1.09) |
| **ALS** |  |  |  |  |
| Low (0-3) | 20/3.91 (5.1) | *Reference* | 19/3.48 (5.5) | *Reference* |
| Moderate (4-5) | 24/5.03 (4.8) | 0.85 (0.47-1.56) | 24/4.48 (5.4) | 0.90 (0.49-1.66) |
| High (6-9) | 15/2.59 (5.8) | 0.94 (0.47-1.87) | 14/2.30 (6.1) | 0.93 (0.46-1.90) |
| Per unit increase | 59/11.53 (5.1) | 1.00 (0.85-1.18) | 57/10.26 (5.6) | 1.02 (0.86-1.20) |

Abbreviations: PY, person-years; IR, incidence rate per 100,000 person-years; HR, hazard ratio; CI, confidence interval; BMI, body mass index

^a^ HR and 95% CI were estimated using Cox model with attained age as the underlying time scale and adjustment for year of birth, BMI, education, physical activity, smoking status, medical history of diabetes and hypertension, and total energy intake

Supplemental Table 5: Consumption of different components of Mediterranean dietary pattern in the Women’s Lifestyle and Health cohort

| Component | Consumption  (Median, g/d) |
| --- | --- |
|  |  |
| Vegetables | 61.95 |
| Fruits and nuts | 136.66 |
| Legumes | 17.54 |
| Cereals | 182.81 |
| Fish and seafood | 22.69 |
| Meat | 84.42 |
| Dairy products | 333.73 |
| M/S ratio | 0.75 |
| Alcohol | 27.64 |

M/S: Monounsaturated fat/saturated fat

Supplemental Table 6: The Strengthening the Reporting of Observational Studies in Epidemiology (STROBE) checklist

STROBE Statement—Checklist of items that should be included in reports of ***cohort studies***

|  | Item No | Recommendation |  |
| --- | --- | --- | --- |
| **Title and abstract** | 1 | (*a*) Indicate the study’s design with a commonly used term in the title or the abstract | X |
|  |  | (*b*) Provide in the abstract an informative and balanced summary of what was done and what was found | X |
| Introduction | | |  |
| Background/rationale | 2 | Explain the scientific background and rationale for the investigation being reported | X |
| Objectives | 3 | State specific objectives, including any prespecified hypotheses | X |
| Methods | | |  |
| Study design | 4 | Present key elements of study design early in the paper | X |
| Setting | 5 | Describe the setting, locations, and relevant dates, including periods of recruitment, exposure, follow-up, and data collection | X |
| Participants | 6 | (*a*) Give the eligibility criteria, and the sources and methods of selection of participants. Describe methods of follow-up | X |
|  |  | (*b*) For matched studies, give matching criteria and number of exposed and unexposed | n/a |
| Variables | 7 | Clearly define all outcomes, exposures, predictors, potential confounders, and effect modifiers. Give diagnostic criteria, if applicable | X |
| Data sources/ measurement | 8* | For each variable of interest, give sources of data and details of methods of assessment (measurement). Describe comparability of assessment methods if there is more than one group | X |
| Bias | 9 | Describe any efforts to address potential sources of bias | X |
| Study size | 10 | Explain how the study size was arrived at | X |
| Quantitative variables | 11 | Explain how quantitative variables were handled in the analyses. If applicable, describe which groupings were chosen and why | X |
| Statistical methods | 12 | (*a*) Describe all statistical methods, including those used to control for confounding | X |
|  |  | (*b*) Describe any methods used to examine subgroups and interactions | X |
|  |  | (*c*) Explain how missing data were addressed | X |
|  |  | (*d*) If applicable, explain how loss to follow-up was addressed | n/a |
|  |  | (*e*) Describe any sensitivity analyses | X |
| Results | | |  |
| Participants | 13* | (a) Report numbers of individuals at each stage of study—eg numbers potentially eligible, examined for eligibility, confirmed eligible, included in the study, completing follow-up, and analysed | X |
|  |  | (b) Give reasons for non-participation at each stage | X |
|  |  | (c) Consider use of a flow diagram | X |
| Descriptive data | 14* | (a) Give characteristics of study participants (eg demographic, clinical, social) and information on exposures and potential confounders | X |
|  |  | (b) Indicate number of participants with missing data for each variable of interest | X |
|  |  | (c) Summarise follow-up time (eg, average and total amount) | X |
| Outcome data | 15* | Report numbers of outcome events or summary measures over time | X |
| Main results | 16 | (*a*) Give unadjusted estimates and, if applicable, confounder-adjusted estimates and their precision (eg, 95% confidence interval). Make clear which confounders were adjusted for and why they were included | X |
|  |  | (*b*) Report category boundaries when continuous variables were categorized | X |
|  |  | (*c*) If relevant, consider translating estimates of relative risk into absolute risk for a meaningful time period | n/a |
| Other analyses | 17 | Report other analyses done—eg analyses of subgroups and interactions, and sensitivity analyses | X |
| Discussion | | |  |
| Key results | 18 | Summarise key results with reference to study objectives | X |
| Limitations | 19 | Discuss limitations of the study, taking into account sources of potential bias or imprecision. Discuss both direction and magnitude of any potential bias | X |
| Interpretation | 20 | Give a cautious overall interpretation of results considering objectives, limitations, multiplicity of analyses, results from similar studies, and other relevant evidence | X |
| Generalisability | 21 | Discuss the generalisability (external validity) of the study results | X |
| Other information | | |  |
| Funding | 22 | Give the source of funding and the role of the funders for the present study and, if applicable, for the original study on which the present article is based | X |

*Give information separately for exposed and unexposed groups.

**Note:** An Explanation and Elaboration article discusses each checklist item and gives methodological background and published examples of transparent reporting. The STROBE checklist is best used in conjunction with this article (freely available on the Web sites of PLoS Medicine at http://www.plosmedicine.org/, Annals of Internal Medicine at http://www.annals.org/, and Epidemiology at http://www.epidem.com/). Information on the STROBE Initiative is available at http://www.strobe-statement.org.
